# A Rare Case of Coronavirus Disease 2019 Survival in an Elderly Patient With Atheroembolic Renal Disease

**DOI:** 10.7759/cureus.14201

**Published:** 2021-03-31

**Authors:** Luigi Cavanna, Elena Zaffignani, Chiara Citterio, Sara De Amicis, Roberto Scarpioni

**Affiliations:** 1 Onco-hematology, Hospital Piacenza, Piacenza, ITA; 2 Nephrology and Dialysis, Hospital Piacenza, Piacenza, ITA

**Keywords:** covid-19, atheroembolic renal disease, cancer, pneumonia, hydroxychloroquine

## Abstract

Atheroembolic renal disease (AERD) is a life-threatening illness. Coronavirus disease 2019 (COVID-19) has a high mortality rate in older patients with comorbidities. We report the case of severe-type COVID-19 in an 82-year-old female with AERD. She was treated with hydroxychloroquine-based therapy and overcame COVID-19. To our knowledge, this is the first report of a patient with AERD and COVID-19 pneumonia who overcame the infection and remains alive and well nine months following infection.

## Introduction

Atheroembolic renal disease (AERD) is caused by the occlusion of small renal arteries from embolized cholesterol crystals arising from ulcerated atherosclerotic plaques. The manifestations include renal failure and systemic atheroembolic disease [1]. The disease develops when atheromatous plaques rupture, releasing cholesterol crystals into the small renal arteries. This can develop spontaneously; however, it usually develops after vascular surgery, catheterization, or treatment with anticoagulants. AERD is associated with poor patient survival [1-3]. Coronavirus disease 2019 (COVID-19) became a pandemic following its initial outbreak in Wuhan, China, in 2019. The majority of reports have indicated that the patients who develop critical illness are old and/or have comorbidities. Indeed, it is well known that patients with comorbid conditions have a high mortality rate and poor outcomes [4,5]. The idea of treating patients with COVID-19 at home was developed in the district of Piacenza in North Italy early in March 2020, and this approach has been publicized [6,7]. We report the first case of an 82-year-old female with AERD who was diagnosed with severe-type COVID-19 and treated at home because she refused hospitalization.

## Case presentation

An 82-year-old Caucasian female patient presented on April 13, 2020, with fever (temperature of 39°C), cough, and dyspnea. She had a history of hypertension, kidney insufficiency, and a creatinine level of 3 mg/dL secondary to AERD, which appeared after surgical treatment for carotid stenosis 10 months earlier. She had undergone hemodialysis six months earlier and was subsequently treated chronically with statins, steroids, amlodipine, and allopurinol for AERD [3] and dicoumarin for atrial fibrillation. The hemodialysis was stopped once her renal function had stabilized. Five years previously, she had a hysteroannessiectomy for endometrial cancer, which was in complete remission, and she was currently in follow-up.

The patient refused hospital admission, and a bedside lung ultrasound (LUS) examination of the anterolateral and posterior chest performed at home using an eight-zone scanning scheme [8] showed bilateral interstitial pneumonia. The patient’s blood pressure was 160/90 mmHg and heart rate 106 beats/minute. A nasopharyngeal swab test was carried out and was found to be positive for COVID-19 with reverse transcription polymerase chain reaction [9]. Her blood oxygen saturation was 92% while breathing room air. The patient was diagnosed with severe-type COVID-19 pneumonia [10]. A blood examination revealed the following: white blood cells (WBC), 12.62 × 109/L; lymphocytes, 1.13 × 109/L; lactate dehydrogenase (LDH), 454 U/L; C-reactive protein (CRP), 4.92 mg/dL; D-dimer, 1,305 ng/mL; and creatinine, 2.46 mg/dL (Table 1).

**Table 1:** Laboratory tests at diagnosis on April 13, 2020. EFU, equivalent fibrinogen units

	Results	Normal range
Red blood cells	4.76	4-5.4 ( × 106/μL)
Hemoglobin	13.3	12-16 (gr/dL)
Hematocrit	43.3	34-46 (%)
White blood cells	12.62	4-10 (×10⁹/L)
Neutrophils	10.54	2-8 (×10⁹/L)
Lymphocytes	1.13	1.5-4 (×10⁹/L)
Platelets	254	150-450 (×10⁹/L)
D-dimer	1,305	≤500 (ng/mL EFU)
Creatinine	2.46	0.6-1 (mg/dL)
C-reactive protein	4.92	0-0.5 (mg/dL)
Lactate dehydrogenase	454	0-247 (U/L)

The patient was treated at home with hydroxychloroquine (HCQ) 400 mg, azithromycin 500 mg, and darunavir/cobicistat 800/150 mg daily. HCQ treatment was incorporated in our regional guideline to treat COVID-19 until May 27, 2020 [11]. All three drugs were delivered for seven days, which was in line with previous recommendations [12], and oxygen was administered. The patient continued her treatment for hypertension and renal disease, which comprised amlodipine 10 mg, atherostatin 40 mg, allopurinol 300 mg, and dicoumarin 2 mg daily. She progressively improved, and after five days, her fever disappeared, her cough was less troublesome, and she reported feeling better. The LUS was repeated every three days until improvement in the second week. The patient gradually recovered from the COVID-19 pneumonia. The patient had ambulatory follow-up every month, and after two months, computed tomography of the chest showed complete resolution of the bilateral pneumonia, and her serum creatinine had stabilized at 2.5 mg/dL. As at the time of writing in January 2021, the patient was alive and well. A blood examination showed improvements in the inflammatory signs: CRP, LDH, WBC, and D-dimer were normalized, and serum creatinine had stabilized at 2.2 mg/dL. To our knowledge, this is the first report of an elderly patient with AERD and COVID-19 pneumonia who overcame the infection and remains alive and well nine months following infection.

## Discussion

AERD occurs in patients with atherosclerotic diffuse plaques and is frequently an iatrogenic disease that occurs after vascular procedures such as angiography or vascular surgery [1]. The patient presented in this report underwent carotid stenosis surgery and was diagnosed with AERD a month later. She subsequently underwent hemodialysis and several hospitalizations for heart and renal failure. It must be emphasized that this disease is generally associated with a poor prognosis and high mortality rates within one year of diagnosis [3]. Therapeutic options are limited. The major causes of death are cardiovascular disease and heart failure. Older age and reduced renal function, which were both present in our patient, are risk factors for death. Severe-type COVID-19 pneumonia also shows a poor prognosis, particularly in older patients with comorbidities [12] like our patient. In addition, our patient had previously undergone a hysteroannessiectomy for endometrial cancer. Reports have indicated that cancer patients receiving active anticancer treatment and cancer survivors like our patient both have a markedly elevated risk of intubation, intensive care unit admission, or death [13].

## Conclusions

We believe that our case is particularly interesting as it demonstrates that a patient with a poor prognosis, notably, with an underlying disease such as AERD, can overcome COVID-19 pneumonia.
